# Asymptomatic Patient with ESKD Presenting with a Purple Peritoneal Dialysis Catheter

**DOI:** 10.34067/KID.0000000772

**Published:** 2025-08-28

**Authors:** Palavi Vaidya, Matthew A. Sparks

**Affiliations:** Division of Nephrology, Department of Medicine, Duke University School of Medicine, Durham VA Health Care System, Durham, North Carolina

**Keywords:** peritoneal dialysis

## Abstract

**Figure:**
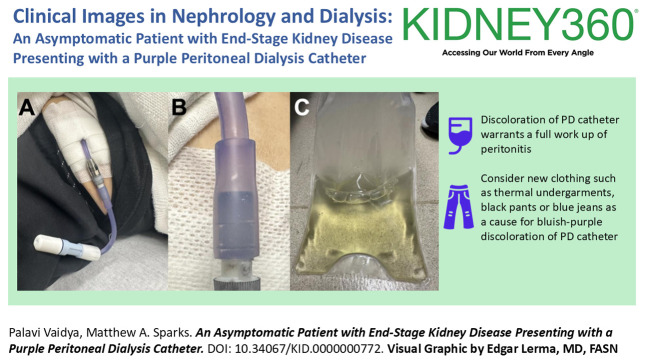

## Case Description

**Figure 1:**
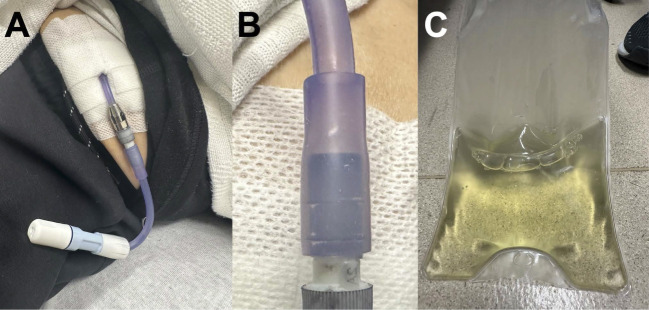
**Purple discoloration of peritoneal dialysis catheter.** (A). PD catheter transfer set with purple discoloration. (B) PD catheter discoloration present proximal to titanium adapter. (C) Clear PD fluid drained after 2-hour dwell. PD, peritoneal dialysis.

A 75-year-old man with hypertension, hyperlipidemia, type 2 diabetes, and kidney failure on peritoneal dialysis (PD) presented with purple discoloration of his PD catheter. He was in his usual health and noticed the discoloration while preparing to bathe. He had no symptoms, such as shortness of breath, chest pain, or fever, and no recent changes to his PD prescription or exposures to new detergents or soaps. He used betadine and chlorhexidine for cleaning (Figure 1).

His vital signs were stable: BP 156/72 mm Hg, heart rate 73 bpm, respiratory rate 16 breaths/min, and temperature 98.2°F. The physical examination was unremarkable except for the purple hue of the PD catheter. A 2-hour dwell showed a total cell count of 6 cells/*µ*l, white blood cell count of 5 cells/*µ*l, no growth on culture, and no organisms on Gram stain. The peritoneal drain fluid was amber and not cloudy. The transfer set was changed as per protocol.

On further questioning, it was revealed that the patient had recently started using a new set of black thermal undergarments, which his wife had washed once before use.

## Discussion

Discoloration of PD catheters from clothing is rarely discussed in the literature. However, a study in Thailand found black-stained catheters in 58 per 1000 continuous ambulatory PD cases. Using a Delphi technique, 127 nurses identified spilled povidone-iodine during transfer set changes as the primary cause. The study concluded that black-stained particles are common contamination linked to care processes.^1^

Purple-stained PD catheters are undocumented in the literature but observed by nephrologists (personal communication). With stable vitals, normal laboratory results, and no peritonitis, we hypothesized that the discoloration was from the patient's new black thermal undergarment. The uniform discoloration suggests dye transfer, supported by clear peritoneal fluid. The patient stopped wearing the undergarment, and the purple hue faded over time.

The Argyle Tenckhoff PD catheter is constructed from silicone rubber, a material known for its dye-resistant properties.^2^ However, disperse dyes, which are frequently used in synthetic fabrics such as polyester, nylon, and spandex, all materials commonly found in thermal underwear, can still stain silicone.^3^

Disperse dyes are polar molecules with anthraquinone or azo groups.^4^ They are designed for synthetic fibers and can interact unexpectedly with silicone. Most are azo dyes (nitrogen-nitrogen double bonds) or anthraquinone dyes (vibrant and stable). Despite silicone's resistance, prolonged contact with these dyes in clothing can cause staining due to their ability to penetrate and bond with synthetic fibers.^5^

In conclusion, the purple discoloration of the patient's PD catheter was likely due to dye transfer from new thermal undergarments. This case underscores the importance of considering external factors such as clothing dyes in PD catheter care. Patients should wash new clothes thoroughly and monitor their PD catheters for changes. Health care providers should educate patients on external contaminants and ensure regular PD equipment inspection and maintenance.

## Teaching Points

- Discoloration of PD catheter warrants a full workup of peritonitis.
- Consider new clothing such as thermal undergarments, black pants, or blue jeans as a cause for bluish-purple discoloration of PD catheter.
